# Soil Acidification Reshapes Microbial Trophic Interactions, with Implications for Plant Responses and Ecosystem Functioning in Tea Plantation Systems

**DOI:** 10.3390/plants15131929

**Published:** 2026-06-23

**Authors:** Seda Bodur, Rasit Asiloglu, Keziban Yazici

**Affiliations:** 1 Department of Horticulture, Faculty of Agriculture, Recep Tayyip Erdogan University, Rize 53300, Türkiye; keziban.yazici@erdogan.edu.tr; 2 Institute of Science and Technology, Niigata University, Niigata 950-2181, Japan; asiloglu@agr.niigata-u.ac.jp; 3 Tea and Tea Products Application and Research Center, Recep Tayyip Erdogan University, Rize 53300, Türkiye

**Keywords:** soil acidification, abiotic stress, environmental filtering, microbial ecology, trophic interactions, plant–soil interactions, protists

## Abstract

Soil acidification is a widespread consequence of intensive agriculture and represents a major abiotic stress affecting plant performance, nutrient availability, and ecosystem functioning. Long-term tea (*Camellia sinensis*) plantations provide model systems of chronic acidification, where sustained low pH imposes strong environmental filtering on soil microbial communities. Although microbial responses to acidification have been extensively studied, research has focused predominantly on bacteria and fungi, leaving other key functional groups, particularly protists, largely overlooked. Here, we synthesize current knowledge on microbial communities in acidified soils and highlight trophic interactions, especially protist-mediated regulation, as a potentially critical but underexplored dimension linking abiotic stress to plant–soil processes. We propose that soil acidification may not only filter microbial community composition but also reshape trophic interactions. Based on evidence from other soil systems, protist-mediated trophic interactions could influence nutrient cycling, pathogen suppression, and ultimately plant responses under stress conditions. Integrating environmental filtering with trophic perspectives provides a conceptual framework for understanding microbiome dynamics in acidified soils. However, direct evidence linking protist-mediated trophic regulation to ecosystem functioning and plant performance in tea plantation soils remains limited and requires experimental validation. We further suggest that these systems provide unique opportunities to investigate how abiotic constraints and biotic interactions jointly shape plant performance. Addressing this gap is essential for advancing predictive understanding of plant–microbiome interactions under ongoing environmental change.

## 1. Introduction

Long-term tea *(Camellia sinensis)* plantations represent some of the clearest agricultural examples of chronic soil acidification, where continuous nitrogen fertilization, high rainfall, and sustained management drive persistently low soil pH [1]. Soil acidification is a widespread consequence of intensive agriculture, with major implications for soil fertility, nutrient availability, and ecosystem sustainability [2,3,4]. Although tea systems are adapted to acidic conditions, excessive and prolonged acidification can exceed optimal ranges, creating a stress gradient that constrains plant performance and alters plant–soil interactions [5,6,7].

Soil pH is one of the strongest determinants of microbial community structure, making chronically acidified systems valuable for understanding how microbial communities respond to long-term abiotic stress [8,9]. Extensive research has therefore examined microbial communities in acidified soils, particularly focusing on bacterial and fungal diversity, community composition, and responses to management practices. These studies have established strong links between soil pH and microbial assembly, and have shown that acidification can alter decomposition, nutrient mineralization, and nitrogen transformations [10,11,12,13]. Global meta-analyses further demonstrate that soil acidification reduces microbial biomass, root biomass, and soil respiration, indicating broad impacts on belowground ecosystem functioning [14]. This body of work provides a solid foundation for understanding microbial responses to low-pH environments and their broader implications for ecosystem multifunctionality in acidic soils [15].

Despite these advances, current knowledge remains largely centered on taxonomic patterns and functional shifts in bacteria and fungi, and therefore provides limited insight into the ecological mechanisms that regulate microbial communities under persistent abiotic stress. These approaches primarily emphasize environmental filtering, while comparatively overlooking the role of biotic interactions in shaping community dynamics and ecosystem processes [16,17]. Microbial communities are structured not only by abiotic constraints, but also by interactions among organisms, including competition, cooperation, and trophic processes, which can regulate population dynamics, nutrient turnover, and ecosystem functioning. Acidification-induced depletion of base cations and accumulation of toxic ions (e.g., Al^3+^ and Fe^3+^) may further restructure soil microbial interactions [14].

Within this underexplored dimension of microbial ecology, protists are especially relevant because they occupy multiple trophic roles and can regulate microbial populations, nutrient cycling, decomposition, and plant-associated processes through direct and indirect interactions [18,19,20,21,22]. Their distribution and activity are also sensitive to soil pH [20,23,24], with soil pH gradients shown to strongly structure micro-eukaryotic communities and alter the relative abundance of major trophic groups [22]. Studies from other soil systems have further shown that environmental changes can alter protist communities and their top-down effects on bacterial and fungal assemblages [25,26]. Together, these findings suggest that soil acidification may influence trophic interactions and associated ecosystem processes, although direct evidence from tea plantation soils remains limited.

**Figure 1:**
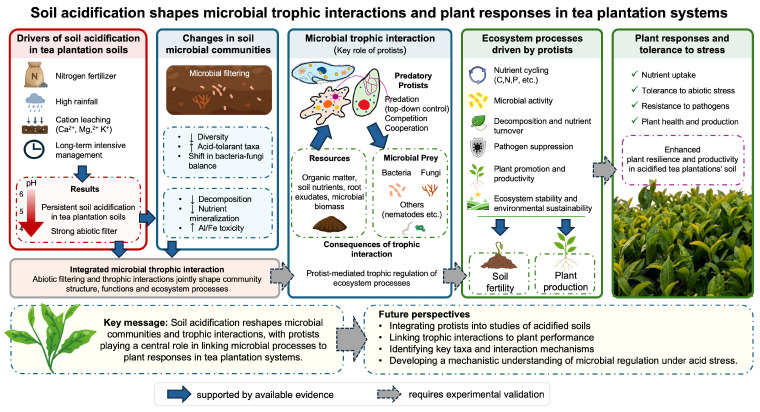
Conceptual framework illustrating the effects of soil acidification on microbial trophic interactions and plant responses in tea (*Camellia sinensis*) plantation systems. Blue arrows indicate relationships supported by available evidence. Grey arrows indicate proposed pathways and conceptual links that require further experimental validation, particularly in tea plantation soils.

While abiotic stress is widely recognized as a major driver of microbial community assembly through environmental filtering, its effects on trophic interactions within microbial food webs remain largely unexplored. We propose that protist-mediated trophic interactions represent a key mechanistic pathway linking soil acidification to plant response. Addressing this gap is essential for developing a more mechanistic understanding of plant–soil systems in acidified environments. To better conceptualize these interactions, we propose a framework integrating environmental filtering and trophic regulation in acidified soils. Tea plantation soils therefore provide valuable systems for investigating how these processes jointly shape microbiome dynamics under chronic acidification (Figure 1).

Soil acidification in long-term tea plantations, driven by continuous nitrogen fertilization, high rainfall, and base cation leaching, acts as a strong environmental filter that reshapes soil microbial community composition. This process reduces microbial diversity and promotes the selection of acid-tolerant bacterial and fungal taxa, leading to altered ecosystem functions such as reduced nutrient mineralization, changes in carbon and nitrogen transformations, and the accumulation of toxic elements (e.g., Al^3+^) [14]. Beyond these abiotic effects, acidification also modifies microbial trophic interactions within the soil food web. Protists, as key microbial predators, regulate bacterial and fungal populations through top-down control, influencing community structure, competition, and biomass turnover. Through the microbial loop, protist grazing enhances nutrient mineralization and facilitates the release of plant-available nutrients, particularly nitrogen and phosphorus, while also contributing to pathogen suppression [27,28,29]. The integration of environmental filtering and trophic regulation results in key ecosystem outcomes under acidic conditions, including improved nutrient availability, stabilization of organic matter decomposition, and maintenance of soil functional processes [14]. These microbial dynamics may also influence plant responses through effects on nutrient acquisition, tolerance to abiotic stress (e.g., low pH and aluminum toxicity), and resistance to pathogens. However, the extent to which these trophic mechanisms contribute to plant growth and productivity in tea plantation soils remains insufficiently understood and requires further investigation.

## 2. Tea (*Camellia sinensis*) Plantations as Model Systems of Long-Term Soil Acidification

Tea (*Camellia sinensis*) plantations are commonly maintained for decades under continuous management, creating long-term agroecosystems in which soil properties develop under sustained anthropogenic pressure. In many tea-growing regions, repeated applications of nitrogen-based fertilizers together with high rainfall drive progressive soil acidification through nitrification and the leaching of base cations [2,4,30,31,32]. Tea plantation soils can exhibit strongly acidic conditions, with reported pH values ranging from 3.14 to 6.39 in major tea-growing regions of Türkiye and from 3.96 to 5.48 across tea-producing provinces in China [1,7]. Long-term tea cultivation is often associated with progressive soil acidification [1]. Although tea is adapted to acidic soils, excessive and prolonged acidification can exceed optimal ranges, leading to constraints on plant performance and alterations in root–microbiome interactions under strongly acidified conditions [33].

The consequences of this acidification extend beyond soil pH. Declining pH alters nutrient availability by increasing the solubility of potentially toxic elements such as aluminum while reducing the availability of essential nutrients such as phosphorus [5,6]. Under acidic conditions, soluble aluminum can further limit phosphorus availability through aluminum–phosphorus interactions and phosphorus fixation, although multiple processes contribute to phosphorus dynamics across soils [34]. Soil acidification may also accelerate the depletion of exchangeable base cations such as calcium and magnesium, contributing to nutrient imbalances and constraints on root nutrient acquisition [35]. These changes can constrain plant performance, modify root functioning, and influence microbial activity, highlighting pH as a central regulator of soil processes in tea plantations. Elevated Al^3+^ availability and associated nutrient imbalances are considered major constraints in strongly acidified tea soils because they can interfere with root development, phosphorus acquisition, nutrient uptake, and overall plant performance [36,37,38,39].

From an ecological perspective, the long-term and relatively stable nature of these acidic conditions creates a consistent selective environment for soil microorganisms. Unlike systems where soil properties fluctuate more strongly through time, tea plantation soils experience sustained abiotic filtering that favors microbial taxa and functions adapted to low pH [10]. This selection pressure can promote distinct microbial communities and alter ecosystem functioning [40].

Unlike many other acidified ecosystems, tea plantations often experience sustained management over decades, recurrent nitrogen fertilization, and relatively predictable acidification trajectories. These characteristics provide a valuable setting for examining how persistent environmental constraints regulate microbial communities, trophic interactions, and soil processes over time.

## 3. Microbial Communities in Tea (*Camellia sinensis*) Plantation Soils: Current Knowledge and Limitations

Microbial communities in tea (*Camellia sinensis*) plantation soils have been increasingly investigated, particularly in chronically acidic systems shaped by long-term management [1,6,11]. Much of this work has focused on bacterial and fungal diversity using high-throughput sequencing approaches, enabling detailed descriptions of community composition across environmental and management gradients [41,42].

These studies have consistently identified soil pH as a major determinant of microbial community structure. Acidification often reduces microbial diversity, restructures community composition, and alters microbial functional potential [13,43,44,45]. Bacterial taxa frequently show strong responses to pH gradients, whereas fungal communities often exhibit greater tolerance to acidic conditions [8,9,10]. Recent evidence further suggests that nutrient-induced acidification can disrupt soil biodiversity–function relationships, thereby altering ecosystem functioning under low-pH conditions [46].

Several studies have also linked acidification to changes in ecosystem functions, including nutrient cycling, nitrogen transformations, microbiome-mediated pathogen suppression, and plant–soil feedbacks [2,47,48,49,50]. These findings indicate that acidification influences not only microbial community structure, but also key ecological processes in tea soils [15]. Studies from agricultural systems have demonstrated that environmental drivers and management practices can substantially restructure soil microbial communities and associated ecosystem functions, reinforcing the importance of environmental filtering in shaping belowground ecosystems [51].

Most evidence from tea plantation systems has been derived from studies focusing on bacterial and fungal communities [52]. Table 1 summarizes representative studies linking tea cultivation, environmental change, and microbiome responses in tea plantation soils.

Despite these advances, research in tea plantation soils has primarily focused on bacterial and fungal communities (Table 1). To our knowledge, no study has specifically characterised protist communities in tea plantation soils using high-throughput sequencing. Studies summarized in Table 1 show that changes in soil physicochemical properties, particularly soil pH, nutrient availability, and soil fertility parameters, are closely associated with shifts in bacterial and fungal community composition and function. These observations suggest that environmental filtering plays a major role in structuring tea soil microbiomes under long-term cultivation and acidification. However, other microbial groups, particularly protists, remain comparatively underrepresented despite their recognized roles in predation, nutrient turnover, and plant-associated processes in other soil systems [18,19,21]. Recent reviews of tea plantation microbiomes similarly highlight that research has largely focused on bacterial and fungal communities, while protists and other microbial groups remain poorly characterized [52]. Although acidification clearly reshapes microbial community composition, current approaches provide limited insight into how interactions among microorganisms regulate ecosystem functioning [58]. Studies from other soil systems have shown that environmental changes can alter protist communities and their top-down regulation of bacterial and fungal assemblages [25,26], suggesting that trophic interactions may contribute to broader microbiome responses under environmental stress. Many ecological patterns observed in acidified soils, including altered nutrient cycling, microbial community dynamics, and plant responses, overlap with established ecological functions of protists (Table 2). These observations suggest that understanding acidified tea soils requires moving beyond taxonomic descriptions toward interaction-based ecological frameworks.

## 4. The Overlooked Role of Trophic Interactions in Acidified Soils

Soil ecosystems are structured through interconnected food webs linking plants, microorganisms, and soil fauna, in which trophic interactions play central roles [67,68]. Similar principles apply within microbial communities, where predators and prey form hidden but functionally important food webs that regulate biomass turnover, nutrient release, and community assembly [17,27]. Among trophic processes, predation is especially important because it can directly alter microbial abundance, competitive balance, and resource availability. By selectively consuming prey, microbial predators may restructure bacterial and fungal communities, stimulate microbial turnover, and enhance nutrient mineralization. These effects can extend to broader ecosystem processes, including carbon and nutrient cycling, pathogen suppression, and plant performance [18,20,29,60,63].

Protists comprise a highly diverse assemblage of eukaryotic microorganisms and perform multiple functional roles in soils, including primary production, decomposition, symbiosis, parasitism, and predation [18,65]. Protists regulate microbial populations through the consumption of bacteria, fungi, other protists, and even nematodes, and promote nutrient conversion [18,19,20,59,61,69]. Experimental evidence further shows that top-down control by protists can be stronger than the bottom-up effects of fertilizers on bacterial community assembly in agricultural soils [22,59]. Indeed, predatory protists are one of the primary factors driving global microbial community divergence [70]. These findings indicate that trophic regulation is not merely theoretical but capable of producing measurable microbiome responses. In addition, the effect of protists extends beyond the plant microbiome. A previous study showed that prey–predator interaction in the rhizosphere soil modulates plant endophytic bacterial communities [64].

These perspectives may be especially relevant in strongly acidified soils, where persistent low pH can constrain community assembly, reduce the range of viable functional pathways, and alter nutrient availability. Under such conditions, trophic interactions may provide additional mechanisms through which microbial populations and ecosystem processes are regulated. For example, by consuming microbial biomass and releasing mineral nutrients through the microbial loop, microbial predators may help sustain nitrogen and phosphorus availability when decomposition and nutrient turnover are slowed [27]. During grazing, protists assimilate only a portion of the nutrients contained in microbial prey and release the excess as plant-available inorganic forms, thereby linking microbial biomass turnover to nutrient recycling. Indeed, several studies have shown that the presence of protists in the rhizosphere soil enhances plant nitrogen uptake [63]. Because fungi often show greater tolerance to acidic conditions than many bacteria, shifts toward fungal dominance may also increase the ecological relevance of mycophagous protists as regulators of fungal biomass and decomposition pathways [10,66]. In addition to low pH itself, soil acidification is frequently accompanied by increased aluminum solubility, altered nutrient availability, and broader changes in soil chemistry. These factors may directly influence protist communities by affecting their survival, activity, trophic interactions, and community composition, although their relative importance remains poorly understood. Therefore, protist responses in acidified soils are likely shaped by multiple interacting environmental factors rather than pH alone.

Together, these observations support a broader conceptual view of acidified soils in which ecosystem outcomes emerge from the combined effects of environmental filtering and biotic interactions, emphasizing the importance of integrating ecological interactions into predictive understanding of belowground processes [71]. Low pH and associated chemical constraints shape community assembly, but the functioning of those filtered communities may depend strongly on trophic regulation, resource flows, and predator–prey dynamics among the organisms that remain. Integrating these dimensions may therefore provide a more complete and mechanistic understanding of how acidified soil ecosystems function under persistent abiotic stress. As these trophic interactions regulate nutrient availability, microbial community structure, and pathogen dynamics, they may provide an important mechanistic pathway linking microbial processes to plant performance in chronically acidified tea (*Camellia sinensis*) soils.

## 5. Trophic Interactions and Plant Responses to Soil Acidification

Soil acidification constrains plant performance by reducing nutrient availability, increasing the solubility of toxic elements such as aluminum, and altering root functioning [5,6]. In addition, acidification can weaken microbiome-mediated pathogen suppression, increasing plant susceptibility to disease [29,48]. Thus, plant responses to low-pH conditions emerge from the combined effects of abiotic stress and biotic pressures in the rhizosphere [33].

Trophic interactions, particularly those mediated by protists, can play a key role in shaping these responses. By selectively grazing on bacteria and fungi, protists stimulate microbial turnover and enhance nutrient mineralization through the microbial loop, potentially sustaining nutrient availability under low-pH conditions [18,27]. At the same time, protist-mediated regulation can promote bacteria with pathogen-suppressive traits, thereby helping to restore microbiome-mediated disease resistance in acidified soils [29,48,62]. Importantly, the effects of protist-mediated trophic interactions are not restricted to the rhizosphere but can extend into plant tissues. Predator–prey interactions in the soil have been shown to reshape endophytic bacterial communities, suggesting that trophic regulation can propagate from soil to plant compartments and directly influence plant-associated microbiomes [64].

These interactions may also contribute to the stability of plant-associated microbial communities by maintaining diversity and functional redundancy under strong environmental filtering [17,24]. Plant traits such as root exudation and species identity further shape microbial assembly and modulate trophic interactions in the rhizosphere, indicating dynamic feedback between plants and microbiomes under acid stress [33]. By regulating bacterial and fungal biomass through predation, protists may influence nutrient mineralization and the release of plant-available nutrients, thereby affecting nutrient acquisition by tea (*Camellia sinensis*) plants under acidified conditions. Integrating trophic interactions into plant–soil frameworks provides a more mechanistic understanding of how plant nutrient acquisition, health, and stress responses are regulated in acidified soils.

## 6. Future Research Priorities in Acidified Soils

Despite recent advances, several important questions about the trophic interactions of protists remain unresolved in acidified soil ecology. Because these systems provide valuable opportunities to study microbial communities under chronic abiotic stress, resolving these gaps could substantially improve predictive understanding of ecosystem functioning under environmental constraints [8,10,72].

Although pH is one of the most important factors controlling trophic interactions [70], direct evidence for how soil acidification alters trophic interactions remains limited. Future studies should examine how low pH influences predator–prey encounter rates, grazing selectivity, and the strength of top-down control across microbial groups. Experimental platforms such as live-cell imaging and controlled pH-gradient microcosms could help quantify these processes under defined chemical conditions.

Trait-based perspectives are also needed to identify which physiological and ecological characteristics confer resilience or sensitivity to acidic conditions. In particular, understanding why some protists persist under low pH through traits such as cyst formation, rapid life cycles, stress tolerance, or flexible feeding strategies would improve predictions of community responses to long-term acidification. Linking these traits with gene expression patterns related to dormancy, osmotic stress, oxidative stress, and recovery responses may provide especially useful mechanistic insight.

Methodological advances now make this progress increasingly feasible. High-throughput sequencing approaches, including bacterial and fungal amplicon surveys together with 18S rRNA profiling of protists, can provide a more complete view of community responses to acidification [73]. However, several methodological challenges remain, including taxonomic uncertainties across many protist lineages, incomplete reference databases, and the limited ability of sequence-based approaches to directly infer trophic interactions and ecological processes. Therefore, integrating sequencing-based surveys with complementary approaches will be important for developing a more mechanistic understanding of protist-mediated processes in acidified soils. Approaches targeting active communities, such as metatranscriptomics, may be especially valuable for identifying metabolically active protists that directly contribute to ecosystem processes [74]. Stable isotope probing and metabolite tracing could further help connect trophic interactions with nutrient flows and biomass turnover. Future studies may also benefit from incorporating ecological stoichiometric perspectives to better understand how acidification-driven changes in nutrient balances influence microbial interactions and nutrient cycling [75].

Experimental tests remain essential for establishing causal relationships. Predator inclusion–exclusion designs, reciprocal community transfers, and manipulative pH-gradient experiments can directly test how abiotic filtering and trophic interactions jointly influence ecosystem functioning. For example, size-fractionation approaches could be used to exclude protists from soil communities, followed by reinoculation with axenic protist cultures to evaluate the effects of trophic interactions on microbial community assembly, nutrient cycling, and plant responses in acidified tea (*Camellia sinensis*) plantation soils [23,59]. In parallel, network inference, structural equation modeling, and trait-based analyses can help disentangle the direct and indirect pathways linking environmental constraints, species interactions, and soil processes [58,72]. Much of the current understanding of tea plantation microbiomes is derived from studies conducted in China, Japan, and India. Future studies across a broader diversity of tea-growing environments will be important for assessing the generality of current observations and identifying context-dependent drivers of microbial community dynamics under long-term acidification [52].

In addition to shaping community structure, soil acidification can also affect the efficiency of protist predation. Protists digest bacterial prey within acidified food vacuoles, where low pH activates hydrolytic enzymes [76,77]. Therefore, environmental acidification may modulate predation efficiency by altering both vacuolar conditions and prey susceptibility. For instance, acid-tolerant bacteria may better withstand intracellular digestion, potentially reduce predation efficiency and shift community composition. However, this hypothesis remains to be experimentally validated.

Applied questions should also receive greater attention. Future research should evaluate whether liming, organic amendments, fertilization strategies, or plant genotype can restore trophic interactions and improve ecosystem functioning in acidified soils [2,4,48]. These approaches may influence soil pH, nutrient availability, microbial activity, and community composition. More broadly, changes in soil fertility status and soil physicochemical conditions can influence nutrient cycling, microbial activity, and microbial-mediated ecosystem functions through their effects on nutrient availability and soil biological activity [78,79]. Previous studies have shown that soil amendments and nutrient management practices, including biochar application and fertilization regimes, can alter protist community composition and functional structure, suggesting that management interventions may influence trophic interactions in acidified soils [80,81]. Long-term sustainability of perennial cropping systems depends on integrated management practices capable of maintaining soil quality, nutrient availability, and biological functioning [82]. Together, these priorities can help move the field from descriptive patterns toward predictive microbial ecology and more sustainable soil management. Future research should also explicitly address how trophic interactions translate into plant-level responses under acidified conditions. Linking microbial community dynamics with plant performance metrics, such as nutrient uptake, growth, and disease resistance, will be essential for developing a more integrated understanding of plant–soil systems under abiotic stress.

## 7. Conclusions

Acidified soils provide valuable systems for understanding how microbial communities respond to persistent environmental stress. The synthesis presented here highlights that microbial regulation under low-pH conditions is shaped not only by strong abiotic filtering, but also by biotic interactions, particularly trophic processes. Explaining ecosystem functioning in these systems therefore requires frameworks that integrate environmental constraints, species interactions, and microbial community dynamics.

Protists deserve particular attention within this perspective because of their capacity to regulate microbial populations, influence nutrient turnover, and affect plant-associated microbiomes. Their sensitivity to environmental gradients further suggests that they may serve as informative indicators and potentially important regulators of microbial processes in acidified soils [18,20,23,24,64,83].

From this perspective, acidified soils—especially long-term systems such as tea (*Camellia sinensis*) plantations—represent valuable field-based systems for testing how abiotic filtering and trophic interactions jointly shape ecosystem processes. Expanding future research beyond bacteria and fungi to include overlooked microbial groups and their interactions may help advance predictive microbial ecology and support more sustainable soil management under ongoing soil acidification. These insights also have important implications for plant–soil interactions and plant performance under acidified conditions [33]. Integrating microbial trophic dynamics into plant-focused frameworks may therefore improve our ability to predict and manage plant responses to abiotic stress. This integrative framework highlights the potential role of protist-mediated trophic interactions as a mechanistic link between soil acidification and plant performance.

## Figures and Tables

**Table 1:** Representative studies linking tea (*Camellia sinensis*) cultivation, environmental change, and microbiome responses in tea plantation soils.

Study Focus	Environmental Change	Microbiome Response	Reference
Comparison of long-term tea cultivation and adjacent natural forest	Long-term tea cultivation reduced soil pH and overall soil health	Reduced bacterial and fungal biomass, lower alpha diversity, and bacterial and fungal communities differed from adjacent forests	[53]
Soil acidification, phosphorus fractions, and phosphorus-cycling microbes	Soil acidification altered phosphorus fractions and availability	Altered phosphorus-cycling microbial communities	[54]
Long-term tea cultivation	Long-term cultivation altered soil properties and promoted acidification	Altered microbial community composition and functional potential	[55]
Large-scale characterization of ancient tea plantation soils	Variation in soil physicochemical properties across tea plantations	Distinct bacterial and fungal community compositions associated with soil characteristics	[41]
Conversion of forestland to tea plantations	Land-use conversion modified soil environmental conditions	Significant shifts in fungal community composition and ecological functions	[15]
Tea–*Pleurotus ostreatus* intercropping	Intercropping modified rhizosphere conditions	Altered fungal diversity and community structure	[42]
Organic fertilizer application	Organic fertilization modified soil conditions	Increased microbial diversity and altered microbial network structure	[56]
Organic management and nitrogen transformation	Organic management altered nutrient dynamics and soil functioning	Enhanced nitrogen transformation and microbial functional activity	[57]

**Table 2:** Conceptual links between soil acidification, protist-mediated trophic roles, and plant–soil functioning in tea (*Camellia sinensis*) plantation soils.

Ecological Dimension	Known Acidification-Related Changes	Potential Protist-Mediated Trophic Roles	Possible Ecosystem and Plant Implications
Microbial community assembly	Reduced bacterial diversity; shifts toward acid-tolerant taxa; altered bacterial–fungal balance [8,10,43]	Altered prey availability; shifts in grazing selectivity; potential restructuring of bacterial and fungal communities through selective predation [17,19,24,59]	Altered microbiome stability and functional redundancy
Nutrient cycling	Reduced decomposition, nutrient mineralization, and nitrogen transformations under low pH [12,13,47]	Sustained microbial turnover through grazing; enhanced nutrient release via the microbial loop; potential support of N and P availability [17,18,27,28]	Changes in nutrient availability and plant nutrient acquisition
Microbial interactions	Disrupted microbial coexistence patterns; altered interaction networks; reduced ecosystem multifunctionality under acid stress [14,17,46,58]	Altered predator–prey encounter rates; shifts in prey preference; changes in the strength of top-down control [17,18,21,60]	Altered microbial network stability and ecosystem functioning
Pathogen suppression	Altered disease-suppressive microbiomes and weakened microbiome-mediated plant protection [48]	Promotion or destabilization of pathogen-suppressive microbial taxa; reshaping of suppressive microbiome functioning [29,61,62]	Changes in disease suppression and plant health
Plant responses	Nutrient limitation, aluminum toxicity, and altered stress responses under acidic conditions [5,6,14]	Modulation of rhizosphere functioning, nutrient uptake, and plant-associated microbiomes [63,64]	Feedbacks on stress tolerance and plant performance
Tea plantation soils	Chronic soil acidification driven by long-term fertilization and high rainfall [1,32]	Selection of acid-tolerant protist taxa; restructuring of microbial food webs under chronic low-pH conditions [19,65,66]	Model system for linking acidification, trophic regulation, and plant performance

## Data Availability

No new data were created or analyzed in this study. Data sharing is not applicable to this article.
